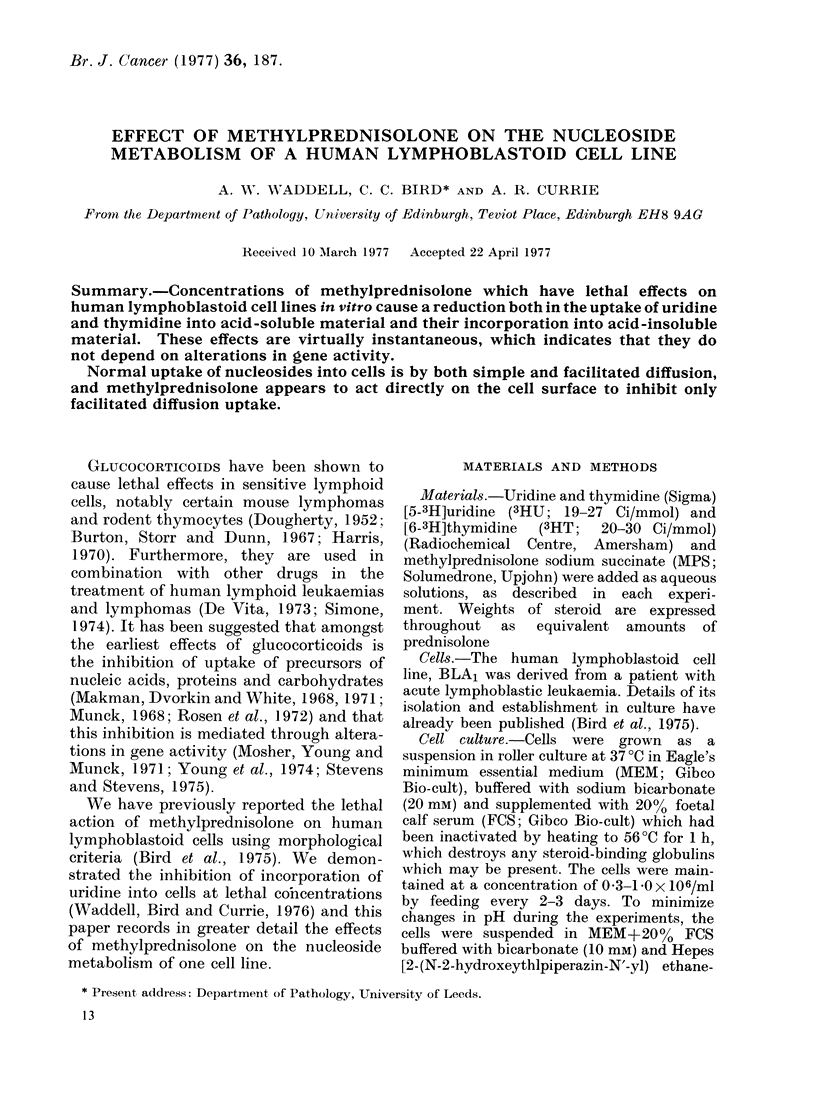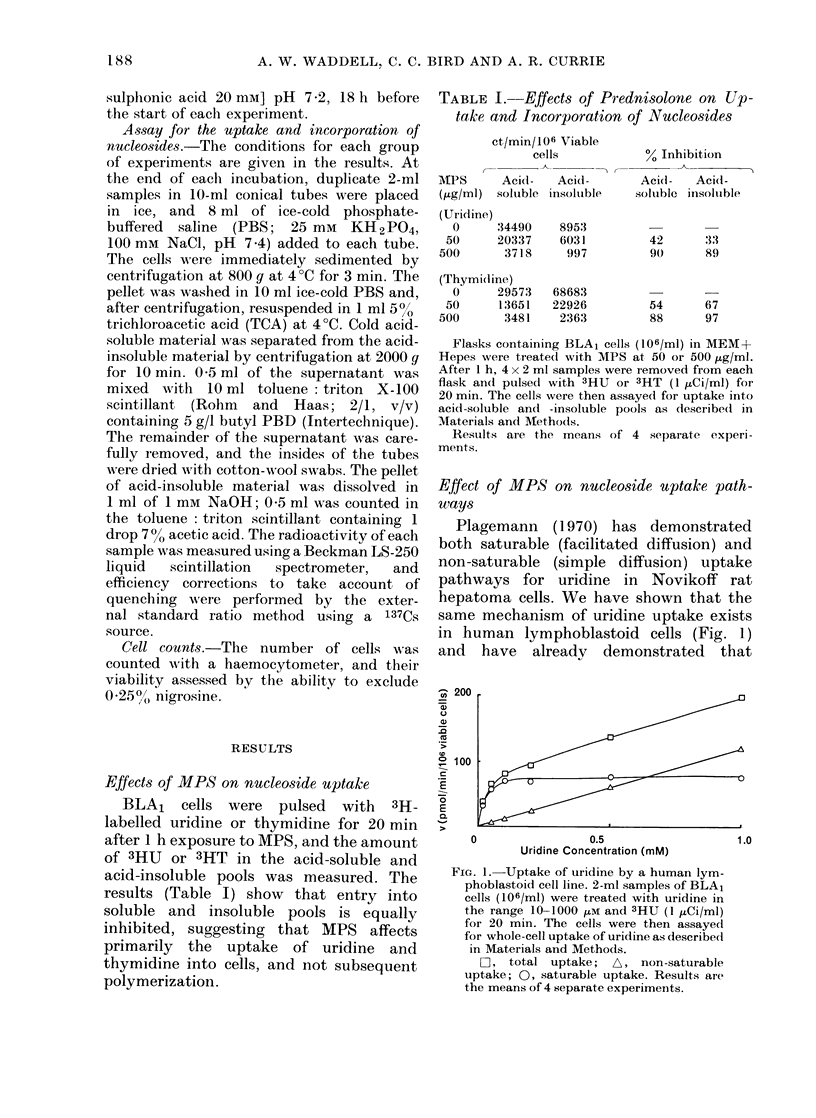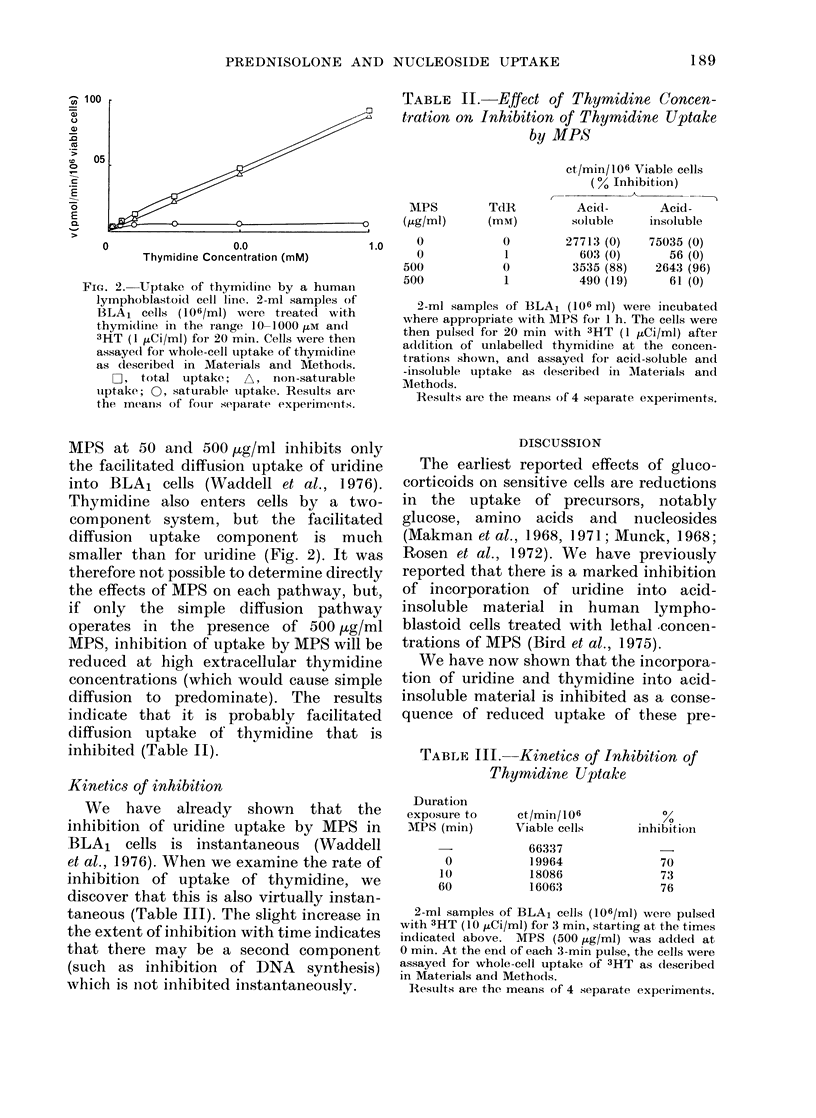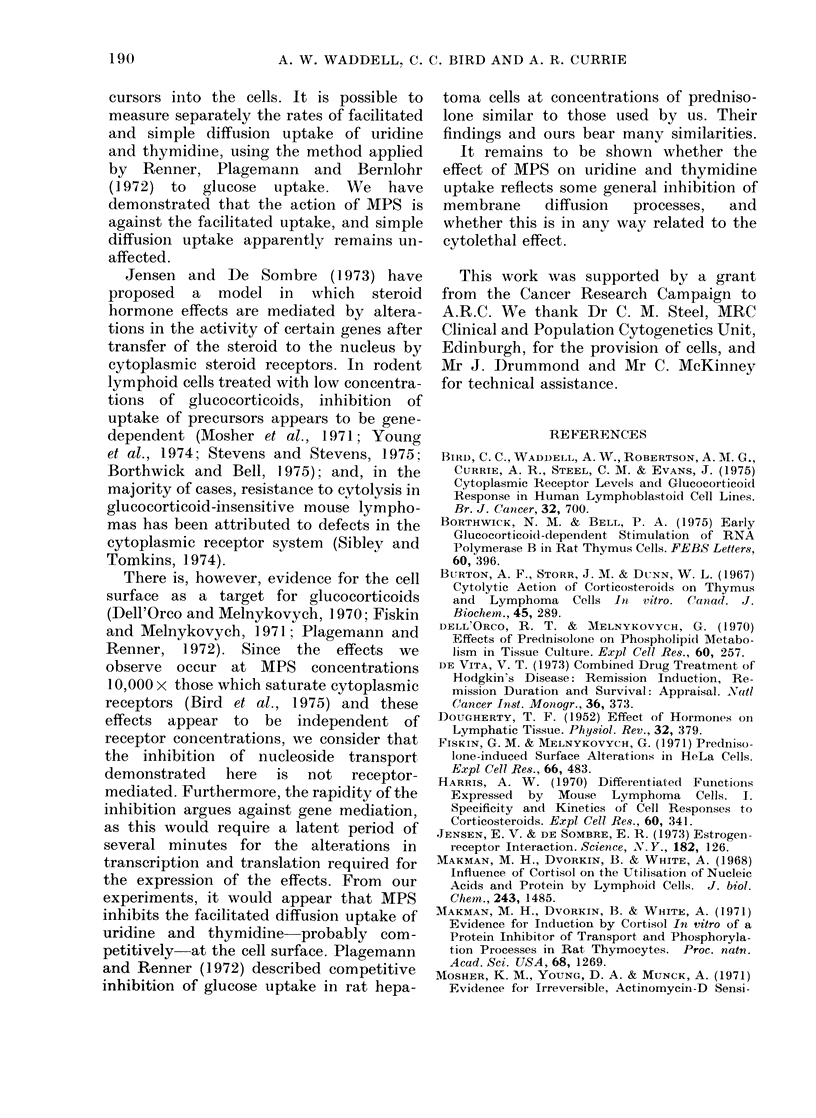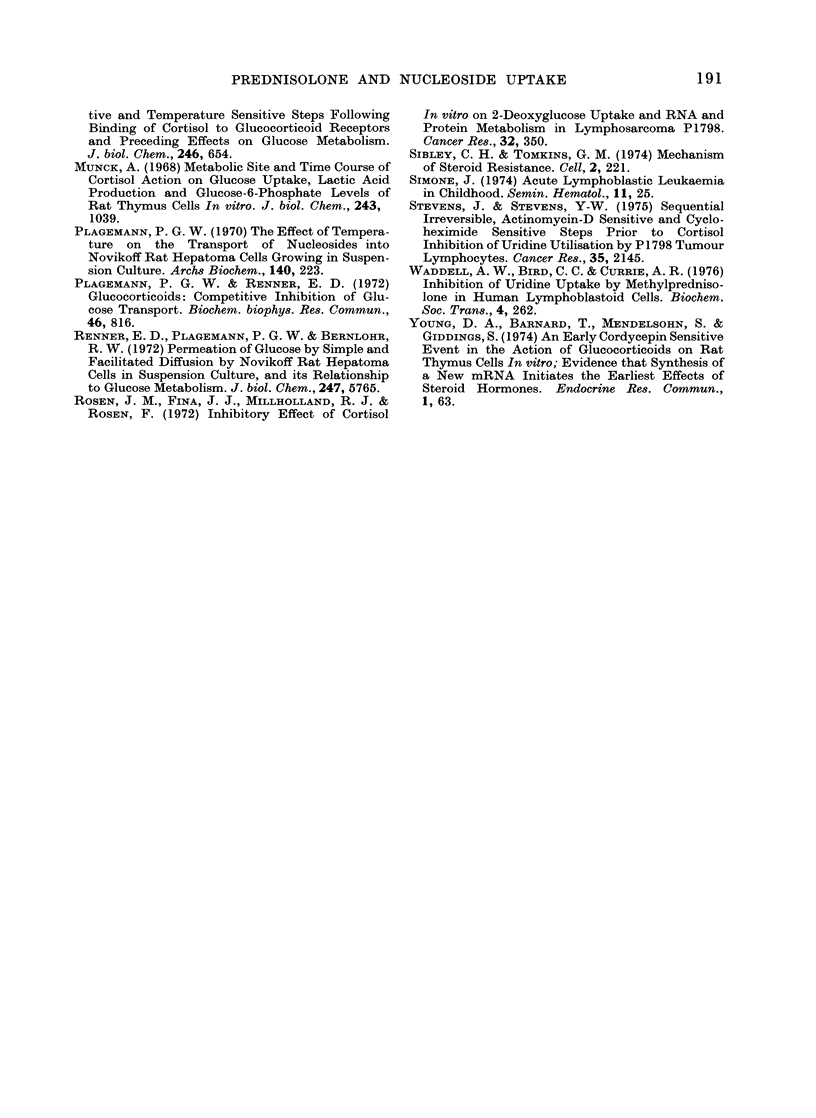# Effect of methylprednisolone on the nucleoside metabolism of a human lymphoblastoid cell line.

**DOI:** 10.1038/bjc.1977.176

**Published:** 1977-08

**Authors:** A. W. Waddell, C. C. Bird, A. R. Currie

## Abstract

Concentrations of methylprednisolone which have lethal effects on human lymphoblastoid cell lines in vitro cause a reduction both in the uptake of uridine and thymidine into acid-soluble material and their incorporation into acid-insoluble material. These effects are virtually instantaneous, which indicates that they do not depend on alterations in gene activity. Normal uptake of nucleosides into cells is by both simple and facilitated diffusion, and methylprednisolone appears to act directly on the cell surface to inhibit only facilitated diffusion uptake.


*Br. J. Cancer* (1977) **36**, 187.

### EFFECT OF METHYLPREDNISOLONE ON THE NUCLEOSIDE METABOLISM OF A HUMAN LYMPHOBLASTOID CELL LINE

A. W. WADDELL, C. C. BIRD* AND A. R. CURRIE

*From the Department of Pathology, University of Edinburgh, Teviot Place, Edinburgh EH8 9AG*

Received 10 March 1977 Accepted 22 April 1977

**Summary.—Concentrations of methylprednisolone which have lethal effects on human lymphoblastoid cell lines *in vitro* cause a reduction both in the uptake of uridine and thymidine into acid-soluble material and their incorporation into acid-insoluble material. These effects are virtually instantaneous, which indicates that they do not depend on alterations in gene activity.**

**Normal uptake of nucleosides into cells is by both simple and facilitated diffusion, and methylprednisolone appears to act directly on the cell surface to inhibit only facilitated diffusion uptake.**

Glucocorticoids have been shown to cause lethal effects in sensitive lymphoid cells, notably certain mouse lymphomas and rodent thymocytes (Dougherty, 1952; Burton, Storr and Dunn, 1967; Harris, 1970). Furthermore, they are used in combination with other drugs in the treatment of human lymphoid leukaemias and lymphomas (De Vita, 1973; Simone, 1974). It has been suggested that amongst the earliest effects of glucocorticoids is the inhibition of uptake of precursors of nucleic acids, proteins and carbohydrates (Makman, Dvorkin and White, 1968, 1971; Munck, 1968; Rosen *et al.*, 1972) and that this inhibition is mediated through alterations in gene activity (Mosher, Young and Munck, 1971; Young *et al.*, 1974; Stevens and Stevens, 1975).

We have previously reported the lethal action of methylprednisolone on human lymphoblastoid cells using morphological criteria (Bird *et al.*, 1975). We demonstrated the inhibition of incorporation of uridine into cells at lethal concentrations (Waddell, Bird and Currie, 1976) and this paper records in greater detail the effects of methylprednisolone on the nucleoside metabolism of one cell line.

### MATERIALS AND METHODS

*Materials.*—Uridine and thymidine (Sigma) [5-$^3$H]uridine ($^3$HU; 19–27 Ci/mmol) and [6-$^3$H]thymidine ($^3$HT; 20–30 Ci/mmol) (Radiochemical Centre, Amersham) and methylprednisolone sodium succinate (MPS; Solumedrone, Upjohn) were added as aqueous solutions, as described in each experiment. Weights of steroid are expressed throughout as equivalent amounts of prednisolone

*Cells.*—The human lymphoblastoid cell line, $BLA_1$ was derived from a patient with acute lymphoblastic leukaemia. Details of its isolation and establishment in culture have already been published (Bird *et al.*, 1975).

*Cell culture.*—Cells were grown as a suspension in roller culture at 37 °C in Eagle's minimum essential medium (MEM; Gibco Bio-cult), buffered with sodium bicarbonate (20 mM) and supplemented with 20% foetal calf serum (FCS; Gibco Bio-cult) which had been inactivated by heating to 56 °C for 1 h, which destroys any steroid-binding globulins which may be present. The cells were maintained at a concentration of $0{\cdot}3–1{\cdot}0 \times 10^6$/ml by feeding every 2–3 days. To minimize changes in pH during the experiments, the cells were suspended in MEM+20% FCS buffered with bicarbonate (10 mM) and Hepes [2-(N-2-hydroxeythlpiperazin-N′-yl) ethane-

* Present address: Department of Pathology, University of Leeds.

sulphonic acid 20 mM] pH 7·2, 18 h before the start of each experiment.

*Assay for the uptake and incorporation of nucleosides.*—The conditions for each group of experiments are given in the results. At the end of each incubation, duplicate 2-ml samples in 10-ml conical tubes were placed in ice, and 8 ml of ice-cold phosphate-buffered saline (PBS; 25 mM $KH_2PO_4$, 100 mM NaCl, pH 7·4) added to each tube. The cells were immediately sedimented by centrifugation at 800 *g* at 4 °C for 3 min. The pellet was washed in 10 ml ice-cold PBS and, after centrifugation, resuspended in 1 ml 5% trichloroacetic acid (TCA) at 4 °C. Cold acid-soluble material was separated from the acid-insoluble material by centrifugation at 2000 *g* for 10 min. 0·5 ml of the supernatant was mixed with 10 ml toluene : triton X-100 scintillant (Rohm and Haas; 2/1, v/v) containing 5 g/l butyl PBD (Intertechnique). The remainder of the supernatant was carefully removed, and the insides of the tubes were dried with cotton-wool swabs. The pellet of acid-insoluble material was dissolved in 1 ml of 1 mM NaOH; 0·5 ml was counted in the toluene : triton scintillant containing 1 drop 7% acetic acid. The radioactivity of each sample was measured using a Beckman LS-250 liquid scintillation spectrometer, and efficiency corrections to take account of quenching were performed by the external standard ratio method using a $^{137}Cs$ source.

*Cell counts.*—The number of cells was counted with a haemocytometer, and their viability assessed by the ability to exclude 0·25% nigrosine.

### RESULTS

### *Effects of MPS on nucleoside uptake*

$BLA_1$ cells were pulsed with $^3H$-labelled uridine or thymidine for 20 min after 1 h exposure to MPS, and the amount of $^3HU$ or $^3HT$ in the acid-soluble and acid-insoluble pools was measured. The results (Table I) show that entry into soluble and insoluble pools is equally inhibited, suggesting that MPS affects primarily the uptake of uridine and thymidine into cells, and not subsequent polymerization.

TABLE I.—*Effects of Prednisolone on Uptake and Incorporation of Nucleosides*

| | ct/min/$10^6$ Viable cells | | % Inhibition | |
|---|---|---|---|---|
| MPS (μg/ml) | Acid-soluble | Acid-insoluble | Acid-soluble | Acid-insoluble |
| (Uridine) | | | | |
| 0 | 34490 | 8953 | — | — |
| 50 | 20337 | 6031 | 42 | 33 |
| 500 | 3718 | 997 | 90 | 89 |
| (Thymidine) | | | | |
| 0 | 29573 | 68683 | — | — |
| 50 | 13651 | 22926 | 54 | 67 |
| 500 | 3481 | 2363 | 88 | 97 |

Flasks containing $BLA_1$ cells ($10^6$/ml) in MEM+Hepes were treated with MPS at 50 or 500 μg/ml. After 1 h, 4 × 2 ml samples were removed from each flask and pulsed with $^3HU$ or $^3HT$ (1 μCi/ml) for 20 min. The cells were then assayed for uptake into acid-soluble and -insoluble pools as described in Materials and Methods.

Results are the means of 4 separate experiments.

### *Effect of MPS on nucleoside uptake pathways*

Plagemann (1970) has demonstrated both saturable (facilitated diffusion) and non-saturable (simple diffusion) uptake pathways for uridine in Novikoff rat hepatoma cells. We have shown that the same mechanism of uridine uptake exists in human lymphoblastoid cells (Fig. 1) and have already demonstrated that

FIG. 1.—Uptake of uridine by a human lymphoblastoid cell line. 2-ml samples of $BLA_1$ cells ($10^6$/ml) were treated with uridine in the range 10–1000 μM and $^3HU$ (1 μCi/ml) for 20 min. The cells were then assayed for whole-cell uptake of uridine as described in Materials and Methods.

□, total uptake; △, non-saturable uptake; ○, saturable uptake. Results are the means of 4 separate experiments.

FIG. 2.—Uptake of thymidine by a human lymphoblastoid cell line. 2-ml samples of $BLA_1$ cells ($10^6$/ml) were treated with thymidine in the range 10–1000 $\mu$M and $^3$HT (1 $\mu$Ci/ml) for 20 min. Cells were then assayed for whole-cell uptake of thymidine as described in Materials and Methods. □, total uptake; △, non-saturable uptake; ○, saturable uptake. Results are the means of four separate experiments.

TABLE II.—*Effect of Thymidine Concentration on Inhibition of Thymidine Uptake by MPS*

| | | ct/min/$10^6$ Viable cells (% Inhibition) | |
|---|---|---|---|
| MPS ($\mu$g/ml) | TdR (mM) | Acid-soluble | Acid-insoluble |
| 0 | 0 | 27713 (0) | 75035 (0) |
| 0 | 1 | 603 (0) | 56 (0) |
| 500 | 0 | 3535 (88) | 2643 (96) |
| 500 | 1 | 490 (19) | 61 (0) |

2-ml samples of $BLA_1$ ($10^6$ ml) were incubated where appropriate with MPS for 1 h. The cells were then pulsed for 20 min with $^3$HT (1 $\mu$Ci/ml) after addition of unlabelled thymidine at the concentrations shown, and assayed for acid-soluble and -insoluble uptake as described in Materials and Methods.

Results are the means of 4 separate experiments.

MPS at 50 and 500 $\mu$g/ml inhibits only the facilitated diffusion uptake of uridine into $BLA_1$ cells (Waddell *et al.*, 1976). Thymidine also enters cells by a two-component system, but the facilitated diffusion uptake component is much smaller than for uridine (Fig. 2). It was therefore not possible to determine directly the effects of MPS on each pathway, but, if only the simple diffusion pathway operates in the presence of 500 $\mu$g/ml MPS, inhibition of uptake by MPS will be reduced at high extracellular thymidine concentrations (which would cause simple diffusion to predominate). The results indicate that it is probably facilitated diffusion uptake of thymidine that is inhibited (Table II).

### *Kinetics of inhibition*

We have already shown that the inhibition of uridine uptake by MPS in $BLA_1$ cells is instantaneous (Waddell *et al.*, 1976). When we examine the rate of inhibition of uptake of thymidine, we discover that this is also virtually instantaneous (Table III). The slight increase in the extent of inhibition with time indicates that there may be a second component (such as inhibition of DNA synthesis) which is not inhibited instantaneously.

### DISCUSSION

The earliest reported effects of glucocorticoids on sensitive cells are reductions in the uptake of precursors, notably glucose, amino acids and nucleosides (Makman *et al.*, 1968, 1971; Munck, 1968; Rosen *et al.*, 1972). We have previously reported that there is a marked inhibition of incorporation of uridine into acid-insoluble material in human lymphoblastoid cells treated with lethal concentrations of MPS (Bird *et al.*, 1975).

We have now shown that the incorporation of uridine and thymidine into acid-insoluble material is inhibited as a consequence of reduced uptake of these pre-

TABLE III.—*Kinetics of Inhibition of Thymidine Uptake*

| Duration exposure to MPS (min) | ct/min/$10^6$ Viable cells | % inhibition |
|---|---|---|
| — | 66337 | — |
| 0 | 19964 | 70 |
| 10 | 18086 | 73 |
| 60 | 16063 | 76 |

2-ml samples of $BLA_1$ cells ($10^6$/ml) were pulsed with $^3$HT (10 $\mu$Ci/ml) for 3 min, starting at the times indicated above. MPS (500 $\mu$g/ml) was added at 0 min. At the end of each 3-min pulse, the cells were assayed for whole-cell uptake of $^3$HT as described in Materials and Methods.

Results are the means of 4 separate experiments.

cursors into the cells. It is possible to measure separately the rates of facilitated and simple diffusion uptake of uridine and thymidine, using the method applied by Renner, Plagemann and Bernlohr (1972) to glucose uptake. We have demonstrated that the action of MPS is against the facilitated uptake, and simple diffusion uptake apparently remains unaffected.

Jensen and De Sombre (1973) have proposed a model in which steroid hormone effects are mediated by alterations in the activity of certain genes after transfer of the steroid to the nucleus by cytoplasmic steroid receptors. In rodent lymphoid cells treated with low concentrations of glucocorticoids, inhibition of uptake of precursors appears to be gene-dependent (Mosher *et al.*, 1971; Young *et al.*, 1974; Stevens and Stevens, 1975; Borthwick and Bell, 1975); and, in the majority of cases, resistance to cytolysis in glucocorticoid-insensitive mouse lymphomas has been attributed to defects in the cytoplasmic receptor system (Sibley and Tomkins, 1974).

There is, however, evidence for the cell surface as a target for glucocorticoids (Dell'Orco and Melnykovych, 1970; Fiskin and Melnykovych, 1971; Plagemann and Renner, 1972). Since the effects we observe occur at MPS concentrations 10,000× those which saturate cytoplasmic receptors (Bird *et al.*, 1975) and these effects appear to be independent of receptor concentrations, we consider that the inhibition of nucleoside transport demonstrated here is not receptor-mediated. Furthermore, the rapidity of the inhibition argues against gene mediation, as this would require a latent period of several minutes for the alterations in transcription and translation required for the expression of the effects. From our experiments, it would appear that MPS inhibits the facilitated diffusion uptake of uridine and thymidine—probably competitively—at the cell surface. Plagemann and Renner (1972) described competitive inhibition of glucose uptake in rat hepatoma cells at concentrations of prednisolone similar to those used by us. Their findings and ours bear many similarities.

It remains to be shown whether the effect of MPS on uridine and thymidine uptake reflects some general inhibition of membrane diffusion processes, and whether this is in any way related to the cytolethal effect.

This work was supported by a grant from the Cancer Research Campaign to A.R.C. We thank Dr C. M. Steel, MRC Clinical and Population Cytogenetics Unit, Edinburgh, for the provision of cells, and Mr J. Drummond and Mr C. McKinney for technical assistance.

### REFERENCES

Bird, C. C., Waddell, A. W., Robertson, A. M. G., Currie, A. R., Steel, C. M. & Evans, J. (1975) Cytoplasmic Receptor Levels and Glucocorticoid Response in Human Lymphoblastoid Cell Lines. *Br. J. Cancer*, **32**, 700.

Borthwick, N. M. & Bell, P. A. (1975) Early Glucocorticoid-dependent Stimulation of RNA Polymerase B in Rat Thymus Cells. *FEBS Letters*, **60**, 396.

Burton, A. F., Storr, J. M. & Dunn, W. L. (1967) Cytolytic Action of Corticosteroids on Thymus and Lymphoma Cells *In vitro*. *Canad. J. Biochem.*, **45**, 289.

dell'Orco, R. T. & Melnykovych, G. (1970) Effects of Prednisolone on Phospholipid Metabolism in Tissue Culture. *Expl Cell Res.*, **60**, 257.

de Vita, V. T. (1973) Combined Drug Treatment of Hodgkin's Disease: Remission Induction, Remission Duration and Survival: Appraisal. *Natl Cancer Inst. Monogr.*, **36**, 373.

Dougherty, T. F. (1952) Effect of Hormones on Lymphatic Tissue. *Physiol. Rev.*, **32**, 379.

Fiskin, G. M. & Melnykovych, G. (1971) Prednisolone-induced Surface Alterations in HeLa Cells. *Expl Cell Res.*, **66**, 483.

Harris, A. W. (1970) Differentiated Functions Expressed by Mouse Lymphoma Cells. I. Specificity and Kinetics of Cell Responses to Corticosteroids. *Expl Cell Res.*, **60**, 341.

Jensen, E. V. & de Sombre, E. R. (1973) Estrogen-receptor Interaction. *Science, N.Y.*, **182**, 126.

Makman, M. H., Dvorkin, B. & White, A. (1968) Influence of Cortisol on the Utilisation of Nucleic Acids and Protein by Lymphoid Cells. *J. biol. Chem.*, **243**, 1485.

Makman, M. H., Dvorkin, B. & White, A. (1971) Evidence for Induction by Cortisol *In vitro* of a Protein Inhibitor of Transport and Phosphorylation Processes in Rat Thymocytes. *Proc. natn. Acad. Sci. USA*, **68**, 1269.

Mosher, K. M., Young, D. A. & Munck, A. (1971) Evidence for Irreversible, Actinomycin-D Sensitive and Temperature Sensitive Steps Following Binding of Cortisol to Glucocorticoid Receptors and Preceding Effects on Glucose Metabolism. *J. biol. Chem.*, **246**, 654.

MUNCK, A. (1968) Metabolic Site and Time Course of Cortisol Action on Glucose Uptake, Lactic Acid Production and Glucose-6-Phosphate Levels of Rat Thymus Cells *In vitro*. *J. biol. Chem.*, **243**, 1039.

PLAGEMANN, P. G. W. (1970) The Effect of Temperature on the Transport of Nucleosides into Novikoff Rat Hepatoma Cells Growing in Suspension Culture. *Archs Biochem.*, **140**, 223.

PLAGEMANN, P. G. W. & RENNER, E. D. (1972) Glucocorticoids: Competitive Inhibition of Glucose Transport. *Biochem. biophys. Res. Commun.*, **46**, 816.

RENNER, E. D., PLAGEMANN, P. G. W. & BERNLOHR, R. W. (1972) Permeation of Glucose by Simple and Facilitated Diffusion by Novikoff Rat Hepatoma Cells in Suspension Culture, and its Relationship to Glucose Metabolism. *J. biol. Chem.*, **247**, 5765.

ROSEN, J. M., FINA, J. J., MILLHOLLAND, R. J. & ROSEN, F. (1972) Inhibitory Effect of Cortisol *In vitro* on 2-Deoxyglucose Uptake and RNA and Protein Metabolism in Lymphosarcoma P1798. *Cancer Res.*, **32**, 350.

SIBLEY, C. H. & TOMKINS, G. M. (1974) Mechanism of Steroid Resistance. *Cell*, **2**, 221.

SIMONE, J. (1974) Acute Lymphoblastic Leukaemia in Childhood. *Semin. Hematol.*, **11**, 25.

STEVENS, J. & STEVENS, Y-W. (1975) Sequential Irreversible, Actinomycin-D Sensitive and Cycloheximide Sensitive Steps Prior to Cortisol Inhibition of Uridine Utilisation by P1798 Tumour Lymphocytes. *Cancer Res.*, **35**, 2145.

WADDELL, A. W., BIRD, C. C. & CURRIE, A. R. (1976) Inhibition of Uridine Uptake by Methylprednisolone in Human Lymphoblastoid Cells. *Biochem. Soc. Trans.*, **4**, 262.

YOUNG, D. A., BARNARD, T., MENDELSOHN, S. & GIDDINGS, S. (1974) An Early Cordycepin Sensitive Event in the Action of Glucocorticoids on Rat Thymus Cells *In vitro;* Evidence that Synthesis of a New mRNA Initiates the Earliest Effects of Steroid Hormones. *Endocrine Res. Commun.*, **1**, 63.